# Evaluation of the degradation profile of biodegradable polymers in a dynamic in vitro model with artificial feline urine

**DOI:** 10.1177/1098612X261418750

**Published:** 2026-01-21

**Authors:** Daiana R Cardoso, Alexandre Barros, André Meneses, Margarida Pacheco, João F Requicha

**Affiliations:** 1 Research in Veterinary Medicine (I-MVET), Faculty of Veterinary Medicine, Lusófona University – Lisbon University Centre, Lisbon, Portugal; 2 Animal and Veterinary Research Center (CECAV), Faculty of Veterinary Medicine, Lusófona University, Lisboa, Portugal; 3 Associate Laboratory for Animal and Veterinary Sciences (AL4AnimalS), Lisboa, Portugal; 4 Department of Veterinary Sciences, University of Trás-os-Montes and Alto Douro (UTAD), Vila Real, Portugal; 5 Hydrumedical SA, Guimarães, Portugal; 6 Animal and Veterinary Research Center (CECAV), University of Trás-os-Montes e Alto Douro (UTAD), Vila Real, Portugal

**Keywords:** Feline urology, poly-p-dioxanone, poly(glycolide-co-caprolactone), poly(glycolide-co-trimethylene carbonate-co-caprolactone), mechanical testing, absorbable stents

## Abstract

**Objectives:**

This study aimed to evaluate the degradation profiles and mechanical properties of three absorbable polymers – poly(glycolide-co-trimethylene carbonate-co-epsilon-caprolactone) (PGTC), poly-p-dioxanone (PDO) and poly(glycolide-co-epsilon-caprolactone) (PGC) – envisioning the development of biodegradable ureteral stents in feline medicine.

**Methods:**

PGTC, PDO and PGC samples were exposed to artificial feline urine circulated through a dynamic system replicating ureteral flow at 38°C for 8 weeks. Degradation was evaluated through weekly measurements of mass loss and qualitative changes. Tensile strength, strain and stiffness were assessed at defined intervals (day 0, weeks 4, 6 and 7).

**Results:**

PGC degraded completely by week 6, showing rapid loss of tensile strength but consistent stiffness. PGTC exhibited gradual degradation until week 8, at which point the material could no longer be handled because of structural weakening, with surface flaking visible microscopically. PDO did not fragment during manipulation or circulation, maintaining tensile strength over 8 weeks, although stiffness fluctuations and brittleness were observed.

**Conclusions and relevance:**

The polymers showed distinct degradation and mechanical behaviours, providing options for different clinical scenarios. PGC, with rapid degradation, may suit short-term applications. PGTC, with gradual degradation and consistent mechanical properties, could serve intermediate applications. PDO, with slower degradation and prolonged tensile strength, appears suitable for longer-term use. These findings represent a step toward developing biodegradable ureteral stents for feline use, potentially simplifying postoperative management and avoiding stent removal. Biodegradable ureteral stents may improve the management of feline ureteral obstructions by eliminating secondary removal procedures. In this in vitro dynamic model, the polymers degraded in a controlled and predictable manner, without accumulation of debris or flow obstruction in the in vitro system. Future studies should assess whether similar behaviour occurs in smaller tubular structures similar to the feline ureter.

## Introduction

The management of feline ureteral obstruction has gained increasing attention in recent years because of the life-threatening nature of the condition and its association with acute kidney injury if left untreated.^1^ Currently, two strategies frequently employed to address ureteral obstructions in cats are non-biodegradable ureteral stents and subcutaneous ureteral bypass (SUB) systems.^2–6^ Both present recognised limitations. Non-biodegradable stents can lead to complications, particularly persistent lower urinary tract (LUT) signs such as haematuria, stranguria, pollakiuria and recurrent infections. Because the risk of infection increases with the duration of stenting, their removal may become necessary, requiring a second surgical procedure.^3,5^ SUB systems are also associated with documented complications. Perioperative issues include clot obstruction, LUT signs, fluid overload, seroma formation and occasional renal injury. Long-term complications are well described and include device obstruction, often related to mineral debris, catheter kinking that may require revision surgery, recurrent urinary infections including pyelonephritis, LUT signs, pain or discomfort, and device migration.^1,2,6,7^ Accordingly, regular imaging and microbiological monitoring are recommended throughout follow-up.^1^ To overcome these limitations, the development of advanced biodegradable stents tailored to feline anatomical and physiological characteristics may offer important benefits, such as biocompatibility with safe degradation into small molecular by-products, improved mechanical properties compared with currently used polyurethane stents, the possibility of incorporating drug delivery systems, avoidance of a second surgery for removal and reduced risk of surface encrustation.^8,9^ An ideal stent would combine ease of insertion, minimal local side effects, low migration risk and natural degradation after fulfilling its function, eliminating the need for removal and avoiding foreign body reactions.^8–10^ Suitable materials are essential for developing a biodegradable ureteral stent adapted to the small diameter of feline ureters. Poly(glycolide-co-trimethylene carbonate-co-epsilon-caprolactone) (PGTC), poly-p-dioxanone (PDO) and poly(glycolide-co-epsilon-caprolactone) (PGC) are widely studied and used in veterinary medicine as synthetic, absorbable polymers and copolymers specifically designed for sutures.^11^

PGTC consists of three monomers known to present different features, namely 72% glycolide for high tensile strength and rapid initial degradation, 14% trimethylene carbonate to enhance flexibility and reduce brittleness, and 14% epsilon-caprolactone to improve elasticity and regulate degradation. This composition enables a gradual, predictable breakdown, with complete absorption occurring within 60–90 days.^11,12^

PDO, a homopolymer from the cyclic monomer p-dioxanone, offers high initial tensile strength and flexibility upon implantation. It degrades through hydrolysis into biocompatible by-products such as carbon dioxide and water, with a moderate degradation rate, retaining approximately 50% of its tensile strength for 3–4 weeks and fully resorbing in 180–210 days. This slower degradation timeline provides extended tissue support, making it ideal for cases requiring prolonged healing, with minimal inflammatory response and high biocompatibility.^12^

PGC is a copolymer made from glycolide (75%) and epsilon-caprolactone (25%). The high proportion of glycolide provides high tensile strength and fast degradation, while epsilon-caprolactone adds elasticity, flexibility and slower breakdown. It degrades through hydrolysis, with a strength retention period of approximately 2–3 weeks. Complete resorption typically occurs at 90–120 days. PGC has excellent biocompatibility, with minimal tissue reaction.^11^

It should be noted that all degradation timelines provided above correspond to manufacturer data from soft tissue implantation studies and do not reflect behaviour in urine, which may alter degradation kinetics.

Although biodegradable ureteral stents are commercially unavailable, preclinical advancements for human application have shown promising results. The main obstacles to clinical validation of current biodegradable ureteral stent designs are uncontrolled degradation rates, difficulty in maintaining mechanical strength and the risk of obstruction from degradation by-products.^13^

This study aimed to identify polymers and copolymers for a new biodegradable ureteral stent, focusing on materials with predictable degradation patterns and proven tissue compatibility in small animals. By evaluating degradation, we sought to identify the most suitable option for ureteral stent design to follow the standard placement technique. This work addresses the current lack of feline-specific biodegradable ureteral stent research, offering a crucial step towards the clinical translation of dynamic in vitro degradation models.

## Materials and methods

This study comprised an in vitro comparative degradation study of the three different polymers in contact with artificial feline urine (AFU).

### Studied materials

The polymers and copolymers used in this study correspond to absorbable synthetic materials whose biocompatibility and tissue absorption have been proven and described by the manufacturers: PGTC (Monosyn 2/0 USP; B Braun), PDO (Monoplus 2/0 USP; B Braun) and PGC (PGC25 2/0 USP; Atramat). Three samples of each material were used, each 490 mm in length and 0.339 mm in outer diameter. The length was selected to provide sufficient material for all planned tests while allowing an additional margin for handling and potential error.

### Preparation of the AFU

AFU was prepared following a method described previously,^14^ with the composition detailed in Table 1. After circulating through the tubes containing the samples, the AFU was collected in reservoirs and continuously recirculated through the circuit, ensuring constant contact between the samples and AFU. The initial pH was measured at 7, and the artificial urine was replaced weekly.

**Table 1:** Composition and concentration of artificial feline urine used in the study

Substance	Chemical formula	Concentration (g/l)
Sodium sulfate	Na_2_SO_4_	2.395
Potassium chloride	KCl	23.945
Sodium oxalate	Na_2_C_2_O_4_	0.039
Sodium nitrate	NaNO_3_	0.065

### Experimental set-up

**Figure 1:**
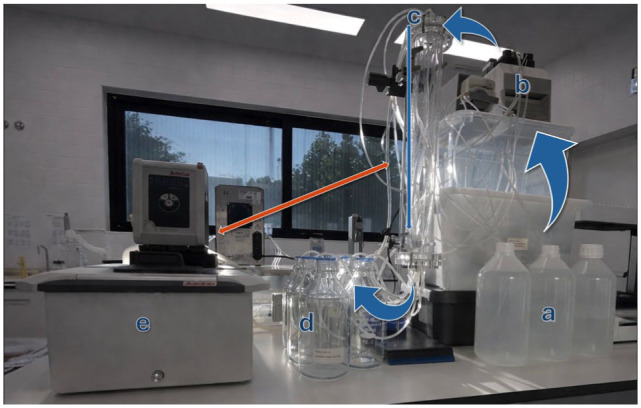
Dynamic experimental system set-up. (a) Artificial urine reservoir; (b) peristaltic pumps (Ismatec Reglo; Cole-Parmer); (c) vessel with the tubes containing each sample; (d) flasks collecting the artificial feline urine (AFU) after passing through the samples; and (e) thermostat (Corio CD Heating Circulator; Julabo) to maintain 38°C in the cylindrical holder with the tubes containing the samples. The orange arrow represents the water circulation between the thermostat and the cylindrical holder; the blue arrows indicate the flow of AFU

An experimental set-up was developed to replicate the dynamic conditions of the ureter for testing nine samples. Each sample was placed inside a plastic tube with an internal diameter of 3.18 mm, which was securely positioned within a cylindrical holder. This diameter was chosen as it represented the smallest tubing size that could be adequately coupled with the experimental set-up. AFU was stored in a common 10 l reservoir and circulated through plastic tubing by peristaltic pumps (Ismatec Reglo; Cole-Parmer). The pumps directed the AFU into the tubes containing the samples, ensuring a steady and consistent flow. After passing through the system, the AFU was directed into reservoirs from which it was continuously recirculated, thereby maintaining constant contact between the samples and the AFU. A thermostat with a water circulating system (Corio CD Heating Circulator; Julabo) was connected to the cylindrical holder, maintaining thermal control. It was set at 38°C to replicate normal feline body temperature. To better simulate feline urinary flow in the ureters, the pumps were set to the minimum possible rate of 0.35 ml/min. A photograph of the dynamic system is presented in Figure 1.

### Degradation

Degradation performance was assessed by weekly weighing: a 20 mm section of each sample was weighed on a precision scale (Precision Balance; Ohaus) at the time of AFU exchange, after the excess AFU had been removed. Samples were considered fully degraded once they could no longer be handled. Macroscopic qualitative assessment was conducted through direct observation every week, focusing on coloration and morphology, categorising samples as intact, fragmented or in particles. Microscopic assessment used a Dino-Lite 5MP digital microscope at × 1 magnification to examine coloration and surface morphology.

### Tensile mechanical analysis

Tensile mechanical analysis of the samples was performed at day 0, week 4, week 6 and week 7 using an Force Measurement ZTA Digital Force Gauge (IMADA) testing machine with a 1.0 kN load cell. These specific time points were selected to capture baseline mechanical properties (day 0), early-stage degradation (week 4) and the critical healing phase (approximately week 6), which corresponds to the reported ureteral healing period in dogs. Week 7 was included to evaluate material performance slightly beyond this physiological window, simulating cases where prolonged stent support might be clinically indicated. Samples 80 mm in length and 0.339 mm in diameter were removed from the tubes, cleared of excess AFU and lightly patted dry with towels before testing. All evaluations were completed within 24 h of removal from AFU. The load was placed midway between the supports with a span of 40 mm. The crosshead speed was 508 mm/min, in accordance with the ASTM F1828–17 standard specification for ureteral stents, which provides guidance on mechanical testing conditions to ensure consistency and comparability of results.^15^ The sample lengths were stretched to the point of breakage, and the peak force was recorded.

### Surface degradation pattern

The assessment of stent surface degradation was performed using an analytical scanning electron microscope (SEM) (JSM-6010LV, JEOL) equipped with energy dispersive x-ray spectroscopy (EDS, INCAx-Act, PentaFET Precision; Oxford Instruments). The accelerating voltages used were 15 eV. SEM analysis was conducted at × 100 and × 300 magnification on day 0, week 2 and week 6. Day 0 was assessed to characterise the initial surface morphology, week 2 to detect early degradation changes and week 6 as a clinically relevant time point corresponding to the expected period of ureteral healing.

### Statistical analysis

Statistical analysis was conducted using Python (version 3.11) with the packages pandas, scipy, statsmodels, seaborn and matplotlib. Descriptive statistics were computed for all numeric variables, including residual weight (g), maximum load (N), tensile strain (%) and Young’s modulus (MPa). Spearman’s rank correlation was used to assess the relationship between experimental time (weeks) and outcome measures for each material. The Kruskal–Wallis test was applied to detect differences in mechanical parameters among materials at each time point. When significance was detected (*P* <0.05), post-hoc Mann–Whitney U-tests were performed with Bonferroni correction.

## Results

### Degradation

**Figure 2:**
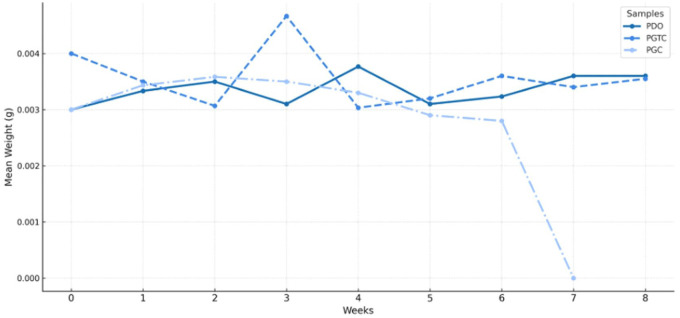
Mean weight (g) of poly(glycolide-co-epsilon-caprolactone) (PGC), poly(glycolide-co-trimethylene carbonate-co-epsilon-caprolactone) (PGTC) and poly-p-dioxanone (PDO) samples over the 8-week incubation period in artificial feline urine under dynamic flow conditions

Figure 2 summarises the evolution of each polymer weight. PGC samples exhibited complete mass loss by week 8, while PDO and PGTC showed limited degradation.

### Macroscopic and microscopic assessment

**Figure 3:**
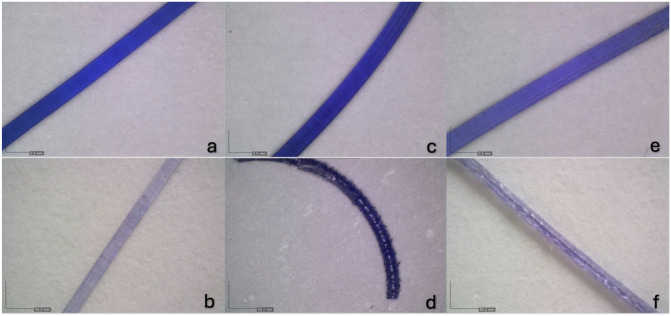
Representative images of the coloration and surface morphology of the studied materials, observed using a DinoLite 5MP digital microscope: (a) poly-p-dioxanone, day 0; (b) poly-p-dioxanone, day 56; (c) poly(glycolide-co-epsilon-caprolactone), day 0; (d) poly(glycolide-co-epsilon-caprolactone), day 42; (e) poly(glycolide-co-trimethylene carbonate-co-epsilon-caprolactone), day 0; (f) poly(glycolide-co-trimethylene carbonate-co-epsilon-caprolactone), day 56

Microscopic evaluation of PDO and PGTC was possible until week 8, and of PGC until week 6. PDO showed a loss of colour, becoming almost completely transparent by week 8 (Figure 3a,b), and increased rigidity during handling. It never fragmented during manipulation or while in the circuit. PGC maintained its colour but began fragmenting within the circuit by week 5, without losing its original shape, and it fragmented easily upon handling. By week 6, all three PGC samples fragmented upon manipulation, and some fragments showed surface flaking (Figure 3c,d). PGTC lost some of its original colour and began showing surface irregularities. By week 8, it could no longer be handled because of structural weakening and displayed surface flaking (Figure 3e,f).

**Figure 4:**
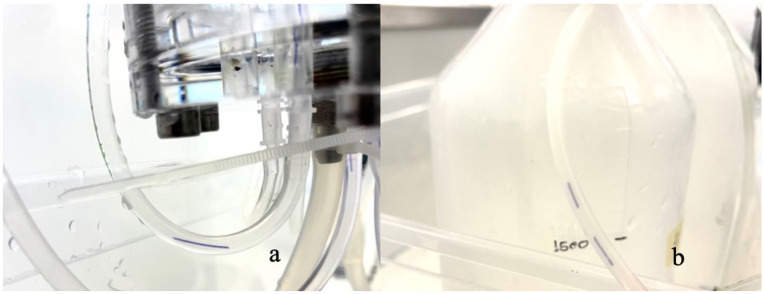
Samples migrating within the dynamic experimental system: (a) poly(glycolide-co-epsilon-caprolactone) sample migrating at week 5; (b) poly(glycolide-co-trimethylene carbonate-co-epsilon-caprolactone) sample migrating at week 8

Within the experimental set-up, both PGC and PGTC fragmented at the distal portion. The fragments were linear, measuring between 3 mm and 5 mm in length. Most fragments disintegrated completely into particles upon attempted removal from the system. Those that remained intact for manipulation under an optical microscope exhibited a scaly surface. The fragments migrated within the circuit without accumulating degradation material or causing any blockage, maintaining a consistently linear movement throughout (Figure 4).

### Maximum load

At day 0 (Table 2), all materials showed the highest maximum load values observed during the study, with mean (±SD) loads for PGC of 8.37 ± 6.48 N, PDO of 9.63 ± 8.83 N and PGTC of 4.80 ± 0.26 N. Over time, PGC demonstrated a progressive decrease, becoming unmeasurable beyond week 4. PGTC also exhibited a decline, reaching a mean of 2.88 ± 2.84 N by week 7. Conversely, PDO retained measurable resistance until week 7, with a moderate decrease from the initial value to 4.03 ± 1.15 N. These trends suggest faster structural degradation in PGC and PGTC compared with PDO.

**Table 2:** Maximum load (N) at different time points for the different materials

Week	PGC	PDO	PGTC
0	8.37 ± 6.48	9.63 ± 8.83	4.8 ± 0.26
4	6.87 ± 6.02	10.63 ± 7.25	4.7 ± 1.55
6	NT	2.67 ± 1.17	3.73 ± 2.65
7	NT	4.03 ± 1.15	2.88 ± 2.84

Data are mean ± SD

NT = not testable; PDO = poly-p-dioxanone; PGC = poly(glycolide-co-epsilon-caprolactone); PGTC = poly(glycolide-co-trimethylene carbonate-co-epsilon-caprolactone)

### Maximum tensile strain

PGC and PGTC showed high initial deformability, with mean values of 34.06% ± 5.88% and 36.10% ± 20.40%, respectively. PDO presented a slightly higher initial strain at 38.05% ± 4.66%. At week 4, PGC and PDO showed a marked reduction in strain capacity, with PDO showing a drastic drop to 4.10% ± 6.68%, while PGTC maintained a high deformability (34.95% ± 4.03%). By week 7, strain capacity was greatly reduced in all groups, with values falling below 8% (Table 3).

**Table 3:** Maximum tensile strain (%) at different time points for the different materials

Week	PGC	PDO	PGTC
0	34.06 ± 5.88	38.05 ± 4.66	36.10 ± 20.40
4	18.91 ± 5.41	4.10 ± 6.68	34.95 ± 4.03
6	NT	4.08 ± 15.55	17.00 ± 6.10
7	NT	4.80 ± 1.70	7.76 ± 1.80

Data are mean ± SD

NT = not testable; PDO = poly-p-dioxanone; PGC = poly(glycolide-co-epsilon-caprolactone); PGTC = poly(glycolide-co-trimethylene carbonate-co-epsilon-caprolactone)

### Young’s modulus

Initially, Young’s modulus values were moderate across materials. By week 4, an increase in mean stiffness was observed in PDO (621.51 ± 613.70 MPa), and to a lesser extent in PGC (373.17 ± 234.57 MPa), while the value for PGTC slightly decreased. At week 7, PGTC showed the highest mean value (405.75 ± 395.11 MPa), suggesting selective retention of a rigid structure despite lower load resistance (Table 4).

**Table 4:** Young’s modulus (MPa) at different time points for the different materials

Week	PGC	PDO	PGTC
0	251.36 ± 180.70	269.57 ± 228.88	177.21 ± 79.45
4	373.17 ± 234.57	621.51 ± 613.70	146.52 ± 32.18
6	NT	151.87 ± 94.03	238.49 ± 137.61
7	NT	194.48 ± 47.05	405.75 ± 395.11

Data are mean ± SD

NT = not testable; PDO = poly-p-dioxanone; PGC = poly(glycolide-co-epsilon-caprolactone); PGTC = poly(glycolide-co-trimethylene carbonate-co-epsilon-caprolactone)

### Surface degradation pattern

**Figure 5:**
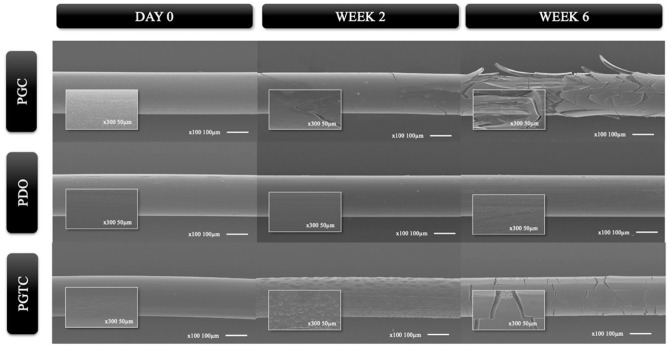
Scanning electron microscopy images showing the surface morphology of three absorbable polymers – PGC, PDO and PGTC – after in vitro incubation for 0, 2 and 6 weeks. For each sample, low-magnification images (100×; scale bar = 100 µm) are displayed in the background, with corresponding high-magnification insets (300×; scale bar = 50 µm) highlighting surface details. PDO = poly-p-dioxanone; PGC = poly(glycolide-co-epsilon-caprolactone); PGTC = poly(glycolide-co-trimethylene carbonate-co-epsilon-caprolactone)

SEM analysis provided a detailed assessment of the surface morphology of the polymers (Figure 5). PGC exhibited a smooth surface at day 0 under both magnifications. By week 2, the first signs of surface degradation began to appear, including cracking and localised delamination. By week 6, PGC showed extensive cracking, fragmentation and a laminated flake-like surface pattern. PDO maintained a smooth and continuous surface morphology throughout the entire evaluation period, without evidence of fissures, delamination or pitting at both 100× and 300×. PGTC showed a smooth surface at day 0. By week 2, early signs of degradation were observed, including the appearance of raised surface structures and micron globules. At week 6, the surface presented defined cracks and mild fragmentation, but without the extensive delamination seen in PGC.

### Comparison of mechanical properties over time across materials

Spearman’s correlation analysis revealed weak and non-significant associations between study week and maximum load, residual weight and Young’s modulus across all materials. In contrast, tensile strain showed a consistent and statistically significant negative correlation with study week. Strong correlations were found for PGTC (ρ = −0.842; *P* <0.001) and PGC (ρ = −0.878; *P* = 0.021), while PDO presented a moderate but significant correlation (ρ = −0.605; *P* = 0.037), indicating a progressive loss of elasticity over time. These results suggest that tensile strain is a more sensitive marker of polymer degradation than mass or stiffness under the tested conditions. A summary of the correlation coefficients and *P* values is presented in Table 5.

**Table 5:** Spearman’s rank correlation coefficients (ρ) and associated *P* values between time and mechanical properties or residual weight for each tested material

Material	Variable	Spearman ρ	*P* value
PGC	Maximum load	0	1
PDO	Maximum load	–0.477	0.117
PGTC	Maximum load	–0.368	0.238
PGC	Tensile strain	–0.878	**0.021**
PDO	Tensile strain	–0.605	**0.037**
PGTC	Tensile strain	–0.842	**<0.001**
PGC	Young’s modulus	0.293	0.573
PDO	Young’s modulus	–0.173	0.591
PGTC	Young’s modulus	0.238	0.457
PGC	Residual weight	–0.273	0.258
PDO	Residual weight	0.221	0.267
PGTC	Residual weight	–0.233	0.253

Statistically significant correlations (*P* <0.05) are shown in bold.

PDO = poly-p-dioxanone; PGC = poly(glycolide-co-epsilon-caprolactone); PGTC = poly(glycolide-co-trimethylene carbonate-co-epsilon-caprolactone)

To explore differences among materials over time, a Kruskal–Wallis test was performed at each time point. A statistically significant difference in tensile strain was found at week 7 (H = 3.857; *P* = 0.049), with a borderline result at week 4 (*P* = 0.051). However, post-hoc pairwise comparisons using the Mann–Whitney U-test with Bonferroni correction did not identify significant differences between specific material pairs, likely due to sample size limitations.

**Figure 6:**
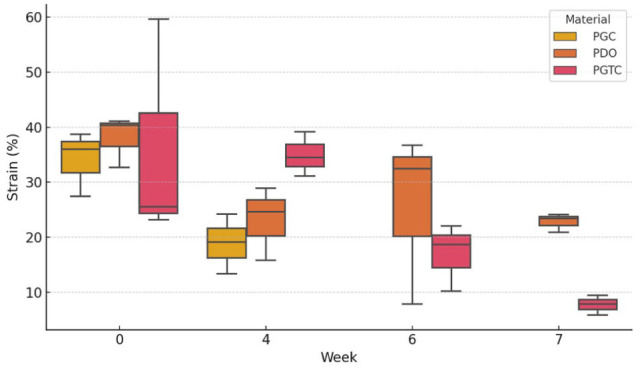
Box plot distribution of tensile strain (%) by material – poly(glycolide-co-epsilon-caprolactone) (PGC), poly-p-dioxanone (PDO) and poly(glycolide-co-trimethylene carbonate-co-epsilon-caprolactone) (PGTC) – and week of incubation

**Figure 7:**
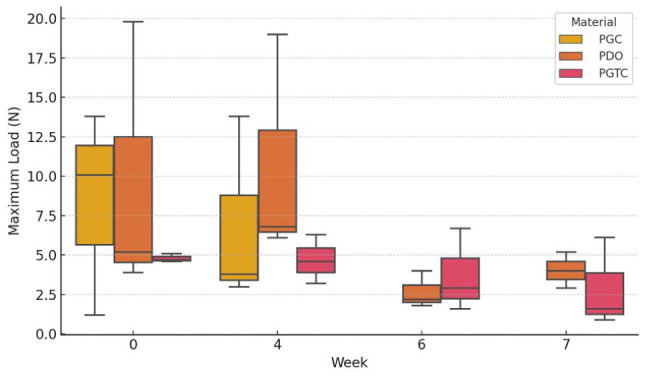
Box plot distribution of maximum load (N) by material – poly(glycolide-co-epsilon-caprolactone) (PGC), poly-p-dioxanone (PDO) and poly(glycolide-co-trimethylene carbonate-co-epsilon-caprolactone) (PGTC) – and week of incubation

The distribution of tensile strain values across weeks and materials is illustrated in Figure 6, clearly reflecting the temporal decline in deformability. The evolution of maximum load, although not statistically significant, showed a downward trend in PGC and PGTC, consistent with macroscopic degradation patterns (Figure 7). Young’s modulus values demonstrated large variability and were not significantly different across materials or time points.

## Discussion

Benign ureteral obstruction in cats remains a major clinical challenge, as it may arise from ureterolithiasis, acquired strictures or pyonephrosis.^6^ Among these causes, calcium oxalate uroliths are particularly relevant, accounting for the vast majority of feline ureteroliths and approximately 90% of urocystoliths. Recent data have shown that the outcome of medical management can be more favourable than previously reported, with an overall success rate of approximately 30% when treatment is maintained for longer periods, with reported success rates of 23% for ureteroliths, 50% for suspected strictures and 50% for pyonephrosis, although interventional procedures, such as ureterotomy,^16^ ureteroneocystostomy,^16,17^ subcutaneous ureteral bypass or stent placement, remain the standard of care for most patients.^18^

Polyurethane ureteral stents have been used for the management of benign ureteral obstructions in cats;^4,16^ however, their clinical application is associated with substantial drawbacks.^5,16^ Reported perioperative mortality rates reach 19%, and complications are frequent.^6^ LUT signs are particularly common,^6,16^ with haematuria reported in 52% of cases, stranguria or pollakiuria in 48% and flank pain in 23%. Long-term complications included stent encrustation and obstruction in up to 26% of cats, often requiring revision surgery. Overall, nearly half (44%) of the cats required additional procedures to address complications, and tolerance is therefore considered limited.^6^ Unlike in human medicine, there are no clear guidelines on the optimal duration of stent placement in veterinary patients, and devices are often maintained until dysfunction occurs, further increasing the risk of adverse outcomes.^5^ Ureteral stents facilitate urinary drainage and passive ureteral dilation, improving short-term patency. However, with increasing indwelling time, progressive mineral encrustation and biofilm accumulation, and in some cases tissue ingrowth, can reduce patency and precipitate dysfunction.^3,19^ Clinical studies specifically describing stent use in conjunction with ureterotomy confirm these limitations: in one series of nine cats, complications included migration (11%), encrustation (11%) and persistent stranguria (11%), with some cases requiring stent removal.^4^ In a larger study of 26 cats, postoperative uroabdomen was reported in 15%, stent migration, fracture or mineralisation necessitated replacement in 19%, and persistent LUT signs were documented in approximately one-third of the population.^5^

In the light of these challenges, we aimed to investigate new ureteral stent materials that offer more predictable and safer degradation patterns, while also being biocompatible and aligned with the typical healing process of the feline ureter. Experimental studies in dogs have shown that ureteral healing is a slow process that involves more than mucosal repair. Mucopolysaccharide content peaked at day 6, collagen deposition at day 14 and tensile strength at day 21; however, by day 42, biochemical parameters had returned to baseline while mechanical strength remained markedly reduced. These findings indicate that ureteral healing requires at least 6 weeks and the ureter remains fragile compared with other tissues, making it essential to select materials that degrade within this time frame.^20^ To our knowledge, no equivalent experimental studies have been performed in cats, and current understanding of feline ureteral healing is therefore largely extrapolated from canine models.

We chose to test three polymers whose use was previously described in cats,^8^ given their advanced characterisation in terms of degradation and biocompatibility. All three degrade primarily through hydrolysis,^21^ and when they are used in a ureteral stent, this degradation process may be further influenced by continuous exposure to urine. The constant flow dynamics and hydration conditions within the urinary tract can accelerate hydrolysis, potentially impacting the stent’s mechanical integrity and lifespan.^13^ In this context, the use of a dynamic system allows a more accurate replication of in vivo conditions. Unlike static models, a dynamic set-up provides a more realistic assessment of how materials behave under continuous exposure to fluid dynamics and body temperature.^10^ To the best of the authors’ knowledge, no previous study in small animals has utilised a similar dynamic system to evaluate biodegradable ureteral stents. This underscores the novelty of the approach and its potential to bridge the gap between in vitro testing and in vivo physiological conditions.

PGC exhibited the fastest degradation, reaching complete mass loss by week 6. This timeline overlaps with the approximate 42-day ureter healing period, raising concerns about its ability to ensure continuous support during the entire process. Mechanically, PGC also showed an early and marked decline in deformability, with mean tensile strain decreasing from 34.06% ± 5.88% at baseline to 18.91% ± 5.41% at week 4, while maximum load similarly declined. In contrast, Young’s modulus remained relatively stable from 251.36 ± 180.70 MPa to 373.17 ± 234.57 MPa within the same period before dropping below measurable levels. This pattern, supported by a significant negative correlation between study week and tensile strain (ρ = –0.878; *P* = 0.0213), indicates progressive loss of elasticity due to early microstructural fatigue while stiffness was initially preserved. Taken together, this profile, characterised by rapid degradation, early decline in deformability and transient preservation of stiffness, suggests that PGC is unsuitable for scenarios requiring sustained mechanical support throughout the ureter healing period. Instead, it would likely be more suitable for cases requiring ureteral stenting in which the ureter maintains adequate structural integrity, such as obstructions caused by ureteroliths or suspected strictures, where the primary goal is to maintain temporary patency rather than to provide mechanical support for tissue healing. However, in both urolithiasis and stricture-associated obstructions, long-term patency after stent dissolution cannot be ensured. The ability of a strictured segment to passively dilate and remain patent after stent removal is not well established, and recurrent obstruction may occur. The duration and mechanism by which patency would be maintained in vivo will ultimately depend on the final stent design and its flow dynamics, aspects that remain under investigation and are beyond the scope of the present degradation study.

PDO samples showed moderate mass loss but remained structurally intact throughout the 56-day experiment, consistent with the manufacturer’s specifications of full resorption within 180–210 days. Mechanically, PDO demonstrated greater resistance to degradation, with maximum load values remaining relatively high until week 7 (from 9.63 ± 8.83 N at day 0 to 4.03 ± 1.15 N). However, its strain capacity declined sharply from 38.05% ± 4.66% to approximately 4–5% after week 4, indicating a transition from elastic to brittle behaviour. This was confirmed by a moderate but statistically significant negative correlation between study week and tensile strain (ρ = –0.605; *P* = 0.0373). Young’s modulus also fluctuated markedly, peaking at 621.51 ± 613.70 MPa at week 4 before decreasing to 194.48 ± 47.05 MPa at week 7, reflecting mechanical inconsistencies and increased rigidity. Taken together, these findings suggest that PDO’s slow degradation and long-lasting structural integrity may be advantageous for stents requiring prolonged support, such as in malignant obstructions. Nonetheless, its rapid loss of elasticity and increased brittleness could limit its performance in the ureter, where flexibility and predictable mechanical behaviour are essential.

PGTC samples exhibited a progressive and consistent degradation pattern. By week 7, they disintegrated easily during handling; by week 8, they could no longer be manipulated. This corresponded with gradual reductions in mean maximum load (from 4.80 ± 0.26 N to 2.88 ± 2.84 N) and tensile strain (from 36.10% ± 20.40% to 7.76% ± 1.80%). In contrast, Young’s modulus increased over time, reaching 405.75 ± 395.11 MPa at week 7 without major fluctuations, reflecting mechanical stability. A strong and statistically significant negative correlation between study week and tensile strain (ρ = –0.842; *P* = 0.0006) confirmed a progressive decline in elasticity, likely related to structural fatigue and microdamage accumulation. Taken together, these results indicate that PGTC combines gradual strength loss, early maintenance of elasticity and stable stiffness, providing predictable degradation and mechanical behaviour. This profile suggests it may be particularly suitable for stents in cases involving ureteral trauma, as its degradation timeline exceeds the typical 42-day healing period and ensures support during tissue repair while maintaining resilience in the early phases of healing.

The SEM observations revealed distinct degradation patterns among the tested polymers, reflecting their differential susceptibility to hydrolytic degradation under in vitro conditions. PGC demonstrated the most rapid and aggressive degradation pattern. Surface fissuring and fragmentation were visible by week 2, with extensive laminar delamination evident by week 6. These morphological alterations are consistent with the early and marked decline in deformability observed in the mechanical tests, with mean tensile strain decreasing from 34.06% ± 5.88% at baseline to 18.91% ± 5.41% at week 4, and with the strong negative correlation between study week and tensile strain (ρ = –0.878; *P* = 0.0213). Together, these findings indicate that PGC rapidly loses elasticity, which limits its ability to provide mechanical support beyond the initial phase of tissue repair and restricts its suitability for applications requiring prolonged structural integrity.

PDO, on the other hand, remained structurally intact throughout the 7-week incubation. This morphological stability aligns with the limited mass loss and the preservation of maximum load observed up to week 7, despite the sharp decline in strain capacity after week 4. Such behaviour is consistent with prior studies in which PDO-based products retained morphology and structural integrity for extended periods in challenging environments,^22^ supporting its potential for applications in which long-lasting support is required, although its increasing brittleness may limit performance in dynamic settings.

PGTC exhibited an intermediate degradation profile. Initially smooth, its surface began to show raised structures and micron globules at week 2, followed by the appearance of cracks and surface fragmentation at week 6, although without the laminar delamination observed in PGC. This progressive morphological evolution mirrors the mechanical findings, which showed gradual reductions in maximum load (from 4.80 N ± 0.26 N to 2.88 N ± 2.84 N) and tensile strain (from 36.10% ± 20.40% to 7.76% ± 1.80%), along with a stable increase in Young’s modulus over time. Such concordance between morphology and mechanics suggests a slower, more controlled degradation mechanism, which may be advantageous in clinical scenarios requiring predictable degradation and sustained support. These observations are consistent with previous studies^23^ in which PGTC SEM showed a two-stage degradation process: early surface integrity followed by gradual hydrolysis that allowed PGTC to maintain stability in the initial stages and minimised debris accumulation, a feature critical for reducing inflammation and microbial colonisation in vivo.

Although neither PGC nor PGTC generated material accumulation or obstruction within the dynamic system, these findings must be interpreted with caution. The tubing used in this model has a larger internal diameter than a feline ureter, which prevents direct extrapolation regarding the risk of obstruction in vivo. Using smaller-diameter tubing would have been more representative of the feline ureter, but this was not technically compatible with the current set-up. Therefore, although the observed linear migration of fragments suggests a potentially favourable fragmentation profile, further studies in systems more closely mimicking the feline ureter are required to confirm the safety of these materials.

Mechanical testing must be interpreted with caution in this context. Unlike sutures or orthopaedic implants subjected to substantial tensile forces, ureteral stents are not designed to provide structural reinforcement under load but rather to maintain luminal patency within a dynamic yet low-stress anatomical environment characterised by subtle peristaltic activity.^22^,^23^ Consequently, parameters such as flexibility, elasticity and controlled degradation are more clinically relevant than ultimate tensile strength.^22^ In this regard, the SEM findings of controlled fragmentation in PGC and PGTC, together with the predictable degradation of PGTC and the prolonged stability of PDO, provide complementary insights for selecting polymers suited to different ureteral stent design requirements.

## Conclusions

Our study indicates that PGC, with its rapid degradation and high initial tensile strength, is the most suitable material for stents designed for short-term use. Its quick loss of structural integrity makes it appropriate for cases without direct trauma to the ureter. In contrast, PDO, with its slower degradation profile and higher residual strength at later time points, is more appropriate for medium- to long-term applications requiring sustained mechanical support, such as malignant obstructions or cases needing prolonged stent functionality. However, its increased brittleness and fluctuations in stiffness over time may reduce its reliability in dynamic environments such as the urinary tract. PGTC, with its balanced and consistent performance, is a versatile option for applications requiring moderate support over an extended period. Its degradation surpasses the typical 42-day healing period, making PGTC suitable for cases involving trauma to the ureter wall, as it would provide reliable support throughout the healing process.

Notably, tensile strain emerged as the most sensitive mechanical parameter for detecting early signs of polymer degradation. Although maximum load and elastic modulus remained relatively stable throughout the incubation period, a consistent and statistically significant decline in strain was observed in all materials, particularly in PGTC and PGC. This progressive loss of tensile deformation capacity indicates that, although the materials retain strength and stiffness, their elastic function deteriorates with time – potentially compromising long-term performance in dynamic or cyclic loading environments. These findings underscore the importance of prioritising the stability of elastic properties when selecting and developing biodegradable polymers for urological applications.

It is important to note that there is no consensus in veterinary practice regarding when to remove a ureteral stent. In humans, the recommended period for removing ureteral stents typically ranges from 48 h to 5 weeks.^24–26^ Removing stents too early may reduce success rates, while leaving them in place for longer than 5 weeks increases the risk of complications such as migration and obstruction.^4,5,10,25^ Furthermore, in human kidney transplantation, the stent should ideally be removed within 3 weeks to reduce the risk of urinary tract infections and other complications.^26^ Given the lack of consensus on the ideal time to remove ureteral stents in veterinary medicine and the fact that this timing may vary depending on the underlying pathology, the present study used as a reference the ureteral healing period of dogs, estimated at approximately 42 days, since equivalent studies in cats are not yet available. Accordingly, mechanical and degradation testing was performed at key time points, with week 4 representing early degradation, week 6 coinciding with the estimated healing window and week 7 extending slightly beyond it to assess material performance when prolonged support may be required.

The linear fragmentation pattern observed for PGC and PGTC, together with the absence of material accumulation or blockage within the experimental system, indicates a potentially favourable degradation profile. However, since the tubing used had a larger internal diameter than a feline ureter, these results should be interpreted with caution and cannot be directly extrapolated to in vivo conditions.

Future studies with larger sample sizes are essential to confirm our findings and reduce the impact of variability on the results. Although the use of a dynamic system that mimics urine flow in the ureter enhances reliability, in vitro conditions cannot fully replicate the complexities of the in vivo environment. Factors such as enzymatic degradation, tissue interaction and physiological stress are critical for determining the clinical performance of these materials. Therefore, future research should include ex vivo and in vivo studies to better understand the degradation profiles and mechanical behaviour in biological systems. In addition, exploring material modifications to optimise degradation rates, tensile properties and biocompatibility may further enhance clinical applicability. Taken together, these findings represent a meaningful step toward the development of biodegradable ureteral stents for feline use, with the potential to simplify postoperative management and eliminate the need for stent removal.
